# Efficacy and safety of orlistat in controlling the progression of prediabetes to diabetes: A meta-analysis and systematic review

**DOI:** 10.1097/MD.0000000000038354

**Published:** 2024-05-24

**Authors:** Zhao Gao, Mengwen Huang, Jiaxin Wang, Huihui Jia, Pin Lv, Jing Zeng, Guixiang Ti

**Affiliations:** a Preventive Medicine Center, China Academy of Traditional Chinese Medicine Guang'anmen Hospital, China Academy of Chinese Medical Sciences, Beijing, China; b China Science and Technology Development Center for Chinese Medicine, State Administration of Traditional Chinese Medicine of The Peoples Republic of China, Beijing, China; c State Key Laboratory of Membrane Biology, School of Medicine, Tsinghua University, Beijing, China; d Department of Orthopaedics, The Fourth Medical Centre, Chinese PLA General Hospital, Beijing, China.

**Keywords:** impaired glucose tolerance, meta-analysis, orlistat, prediabetes

## Abstract

**Background:**

The aim of this study is to examine the impact of the Orlistat on glucose levels and glucose tolerance in individuals with prediabetes, as well as assess its efficacy and safety in preventing the progression to diabetes.

**Methods:**

For achieving the appropriate randomized controlled trials, we enrolled the public datas from the following electronic databases: The Cochrane library, Embase, China National Knowledge Infrastructure, VIP, Wan-Fang, and China Biology Medicine disc. The article focused on the orlistat intervention of glucose tolerance and glycemic status in prediabetic patients. We restricted the publication time from the creation to May 2023.

**Results:**

Six subjects were included in the study, with a total of 1076 participants (532 in the control group vs 544 in the experimental group). The results indicated that the orlistat can reduce the fasting blood glucose [relative risk (RR) = −2.18, 95% confidence intervals (CI) (−2.471, −1.886)], as well as the 2 hour postprandial blood glucose [RR = −1.497, 95% CI (−1.811, −1.183)]. Furthermore, it can prevent the impaired glucose tolerance patients to type 2 diabetes mellitus [RR = 0.605, 95% CI (0.462, 0.791)], and reversal the impaired glucose tolerance [RR = 2.092, 95% CI (1.249, 3.503)].

**Conclusions:**

In prediabetic people, the orlistat can control weight, reduce the fasting blood glucose and the 2 hour postprandial blood glucose, and then delay the progression of diabetes. However, due to the quantitative restrictions, additional high-quality study needs to be conducted to improve the reliability of the results.

## 1. Introduction

The global prevalence of diabetes has increased to 9.3% in 2019 and is projected to rise to 10.2% by 2030.^[1]^ It is estimated that about 4.2 million deaths occurred from diabetes,^[2]^ at the same time, there is a huge global health expenditure on diabetes,^[3]^ from $966 billion in 2021 and is expected to reach $1054 billion in 2045. The widespread prevalence of diabetes has imposed a significant socioeconomic burden on the world, and the outlook remains bleak. It is imperative to mitigate the prevalence of diabetes and regulate the diabetic population. The prevalence of diabetes in China reached 12.4% in 2018,^[4]^ which is higher than the global average, and it continues to exhibit an increasing trend. In China in 2018, the prevalence of prediabetes mellitus (PDM) among patients reached as high as 38.1%.^[1]^ Among them, approximately 10% to 15% of patients with impaired glucose tolerance (IGT) progress to type 2 diabetes mellitus (T2DM) annually.^[2]^ The prediabetes stage represents impaired blood glucose regulation prior to the onset of T2DM. Prediabetes includes 2 states: impaired glucose tolerance and impaired fasting glucose. It serves as an significant reservoir for T2DM development and is also a crucial phase for restoring normal blood glucose levels. Therefore, PDM has emerged as an crucial stage for prevention and warrants attention. Significantly delaying the progression of PDM patients to T2DM can effectively reduce the diabetic population. Currently, pharmacological and lifestyle interventions are the primary approaches for managing prediabetes. Among these, lifestyle interventions hold greater significance. Evidence-based guidelines recommend a limited number of drugs such as metformin, acarbose, and thiazolidinediones.^[5]^ Furthermore, there is evidence suggesting that orlistat may also impede the progression of diabetes in patients with PDM, although its long-term efficacy requires further validation.^[6]^ With regards to the low adherence to lifestyle interventions, it is imperative to explore more effective alternatives to medications. One of the primary objectives of lifestyle interventions is weight loss, and orlistat has offers a straightforward pharmacological approach as well as an adjunctive measure for improved weight management.^[7]^

The research team aimed to conduct a systematic review and meta-analysis of randomized controlled trials to evaluate the efficacy and safety of orlistat in preventing the progression from PDM patients to diabetes.

## 2. Methods

### 2.1. Search strategy

We conducted a comprehensive search of multiple databases including PubMed, Embase, The Cochrane Library, Google Scholar, China Biology Medicine, China National Knowledge Infrastructure, VIP, and Wan-Fang using the following search terms: “Orlistat,” “Xenical,” “Alli,” (“Xenical,” “Alli” are the former trade name of Orlistat, We used as many search terms as possible to find the as full literature as possible.) “prediabetic state,” “prediabetes,” “tetrahydrolipstatin,” “impaired glucose tolerance,” and “impaired glucose regulation.” The database was searched up until May 2023.

### 2.2. Inclusion criteria

All participants must meet the following inclusion criteria: ① be over 18 years of age, and ② have been diagnosed with impaired glucose tolerance based on oral glucose tolerance test (OGTT) results, defined as fasting blood glucose < 7 mmol/L and 2-hour postprandial blood glucose levels between 7.8 mmol/L and 11.1 mmol/L.^[5]^

All the data included in this study are derived from randomized controlled trials, and all the literature cited is published research that excludes unpublished clinical conference proceedings. This study does not impose any restrictions on the race, gender, age, or disease duration of the trial participants. The literature experimental group in this study received orlistat intervention, with a daily dose of 360 mg in 5 articles, 240 mg in 1 article, and administered orally in 6 articles. The control group underwent lifestyle intervention with either placebo or oral metformin. The dosage and duration of the study were unrestricted. Primary outcome measures included control rate of impaired glucose tolerance and incidence of diabetes, while secondary outcome measures comprised 2 hour postprandial blood glucose, fasting blood glucose, body mass index, and occurrence of adverse events.

### 2.3. Exclusion criteria

The following issues were identified in the study: ① unclear diagnosis of IGT among subjects. ② Application of other interventions in the experimental group. ③ Lack of clarify regarding subject glucose tolerance status in the experimental outcome. ④ Presence of data duplicates within the article. ⑤ Non-RCT study design.

### 2.4. Quality assessment

The quality of the literatures was assessed using the Jadad rating scale, which evaluated random sequence generation, randomization concealment, blinding, dropouts, and lost visits (Table 1). All 6 experiments were randomly assigned with varying degrees of randomization concealment. Four studies utilized placebo-blinded experiments while 3 studies described the reasons or nodes of subject withdrawal. The limitations of inadequate blinding and incomplete data may impact the evaluation outcomes, while the small size of individual experimental subjects could compromise the precision of the experiment. Characteristics of the included studies are shown in Table 2.

**Table 1:** The Jadad grading scale.

Study	Randomization	Allocation concealment	Blind method	Withdrawal	Score
Steven B (2000)^[8]^	Unclear 1	Unclear 1	Adequate 2	Description 1	5
Torgerson JA (2004)^[9]^	Adequate 2	Unclear 1	Adequate 2	Description 1	6
Ying Han (2004)^[10]^	Unclear 1	Unclear 1	Adequate 2	Undescribed 0	4
Ke Zhang (2007)^[11]^	Unclear 1	Unclear 1	Inadequate 0	Description 1	3
Dixon AN (2008)^[12]^	Unclear 1	Unclear 1	Inadequate 0	Description 1	3
Dong Liu (2018)^[13]^	Adequate 2	Unclear 1	Adequate 2	Undescribed 0	5

**Table 2:** Characteristics of the included studies.

Study	County	Sample size	Outcome indicators	Experimental time
Steven B (2000) ^[8]^	America, Europe	120	Sugar tolerance, FBG	577 days
Jarl S (2004)^[9]^	Sweden	694	Sugar tolerance	4 years
Ying Han (2004)^[10]^	China	98	Sugar tolerance, FBG, 2hBG, BMI	1 year
Ke Zhang (2007)^[11]^	China	64	Sugar tolerance, FBG, 2hBG, weight	5 months
AN Dixon (2008)^[12]^	Britain	40	FBG, 2hBG, BMI, weight	1 year
Dong Liu (2018)^[13]^	China	60	Sugar tolerance, FBG, 2hBG, BMI, HbA1c	6 months

2hBG = 2 hour postprandial blood glucose, BMI = body mass index, FBG = fasting blood glucose, HbA1c = glycosylated hemoglobin, type A1C.

### 2.5. Data extraction

The literature screening, data extraction, evaluation, and proofreading were independently conducted by 2 researchers. Data collection primarily encompassed basic research information such as article title, publication date, author; study methods including subject characteristics, sample size, intervention measures, randomization method, intervention time, blindness; outcome indicators comprising names of each indicator and corresponding data results along with loss to follow-up and withdrawal.

### 2.6. Statistical analysis

Meta-analysis was conducted using Stata16.0 software. The statistics were presented as relative risk (RR) with corresponding 95% confidence intervals (95% CI). Heterogeneity among the included studies was assessed by the χ2 test, and a fixed-effect model was used If heterogeneity between studies was low (*I*^2^ < 50%, *P* > .1), while a random-effect model was exploited for high heterogeneity (*I*^2^ > 50%, *P* < .1). Publication bias was evaluated through funnel plot analysis. A statistically significant difference was considered when *P* < .05.

### 2.7. Registration

This present article has been duly registered on the Prospero website under registration number CRD42023426494. This study does not involve ethics committee approval, because this study is a secondary data analysis using data published in databases, and does not involve biological specimens and human ethics.

## 3. Results

### 3.1. Literature screening results

**Figure 1.**
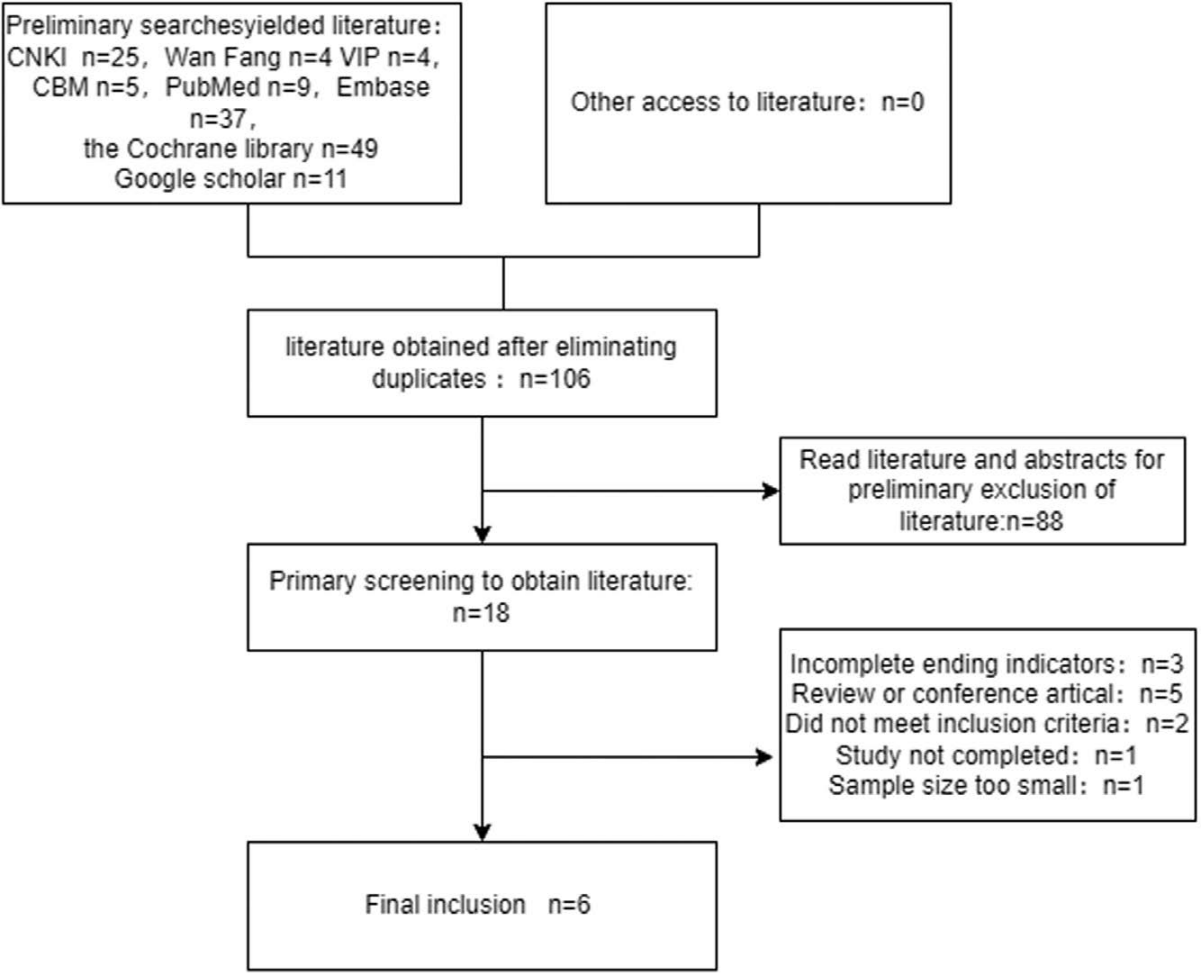
Flow chart of literature screening for patients with abnormal glucose tolerance in prediabetes (CBM = China Biology Medicine; CNKI = China National Knowledge Infrastructure).

The initial search of this study yielded a total of 133 articles, with China National Knowledge Infrastructure contributing 25, Wan-Fang providing 4, VIP offering 4, PubMed supplying 9, Embase presenting 37, Cochrane library contributing 49, google scholar 11 and China Biology Medicine adding another 5. After removing duplicates, we were left with a pool of 106 articles to review. Following an assessment of titles and abstracts, we excluded a further 88 articles from consideration. A initial screening process resulted in the selection of just 18 articles for full text review. Ultimately twelve studies were excluded after reading their full text leaving us with 6 studies that met our inclusion criteria as shown in Figure 1.^[8–13]^

### 3.2. Basic characteristics and quality assessment

This study included a total of 1076 PDM patients aged over 18 years, with sample sizes varying widely across experiments from hundreds of larger cases to dozens of smaller cases. Participants were recruited from China, the United Kingdom, Sweden, and the United States.

The orlistat utilized in this study was a state-approved medication with treatment durations ranging from 6 to 48 months, and the dosage administered during the study ranged from 240 mg to 360 mg per day. All 6 experiments adhered to identical diagnostic criteria, whereby IGT could be diagnosed between 7.8 to 11.0 mmol after 2 hours of OGTT. The orlistat utilized in this study was a state-approved drug administered for a duration of 6 to 48 months, with doses ranging from 240 mg to 360 mg/day. All 6 experiments adhered to the same diagnostic criteria, which involved diagnosing IGT between 7.8 to 11.0 mmol after 2 hours of OGTT experimentation. Five studies^[8–11,13]^ reported on the conversion rate of IGT to T2DM, 4^[8,10,11,13]^ reported on the rate at which IGT returned to normal levels, and 4^[10–13]^ reported on the results of the 2-hour postprandial test.^[8,10–13]^

Five studies reported the conversion of IGT to T2DM, four reported the rate at which IGT was torsioned to normal, 4 reported 2-hour postprandial blood glucose levels, and 5^[8,10–13]^ reported fasting blood glucose levels.

### 3.3. Meta-analysis results

#### 3.3.1. Prevalence of diabetes mellitus

**Figure 2.**
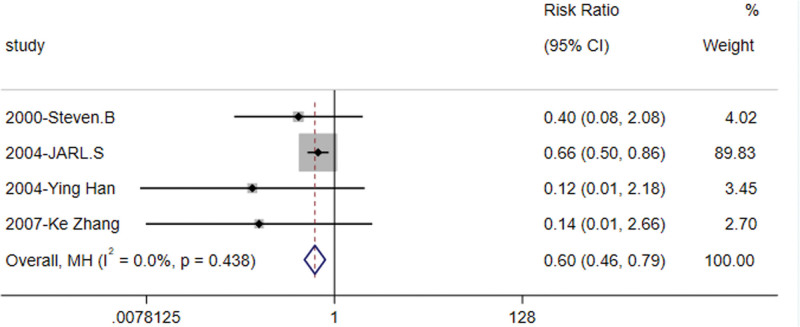
Forest plot of diabetes incidence with the orlistat intervention versus the control group.

A total of 5^[8–11,13]^ articles reported the conversion rate to diabetes in patients with orlistat intervention for IGT, comprising 1036 subjects. The heterogeneity test results showed little heterogeneity between studies (*I*^2^ = 0, *P* = .438), thus a fixed effects model was used for meta-analysis which revealed statistically significant differences between the 2 groups [RR = 0.605, 95% CI (0.462, 0.791), Z = −3.677, *P* = .000] (Fig. 2). The findings indicate that the incidence of T2DM was lower in patients receiving orlistat intervention compared to those in the control group, and orlistat demonstrated efficacy in managing the progression to T2DM among individuals with IGT.

#### 3.3.2. Control rate of abnormal glucose tolerance

**Figure 3.**
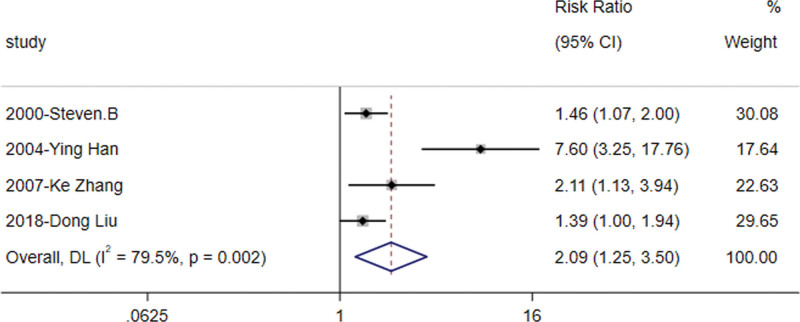
Forest plot of the abnormal glucose tolerance control rate of the orlistat intervention versus the control group.

Four studies^[8,10,11,13]^ reported data on the reversal of IGT status to normal glucose tolerance in a total of 342 subjects. There was high heterogeneity (*I*^2^ = 79.5%, *P* = .002), and a random effects model was used for meta-analysis which showed statistically significant results [RR = 2.092, 95% CI (1.249, 3.503), Z = 2.806, *P* = .005] (Fig. 3). The finding indicate that orlistat significantly enhances the rate of reversion to normal glucose tolerance in patients with IGT compared to the control group. This suggests that orlistat is an effective intervention for preventing further progression to diabetes and reversing IGT.

#### 3.3.3. Fasting blood glucose

**Figure 4.**
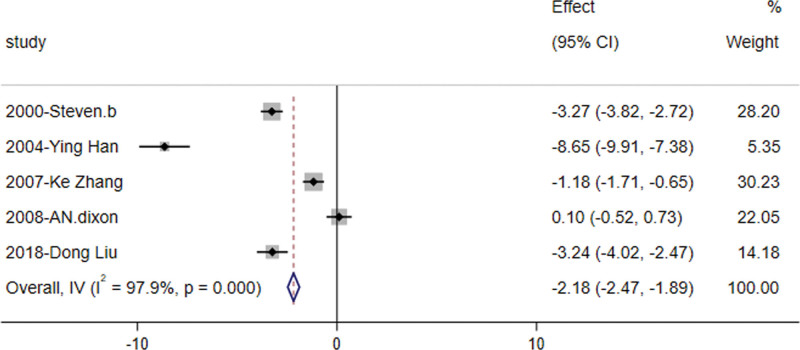
Forest plot of fasting blood glucose levels in the orlistat intervention versus control group.

The analysis revealed that orlistat significantly decreased fasting blood glucose levels in patients with IGT compared to controls [RR = −2.18, 95% CI (−2.471, −1.886), z = −14.588, *P* = .000] (Fig. 4). This finding was based on 5 studies^[8,10–13]^ involving 382 subjects reporting on fasting blood glucose in the IGT population, which exhibited high heterogeneity (*I*^2^ > 50%) and were analyzed using a random-effect model.

#### 3.3.4. Blood glucose for 2 hour postprandial blood glucose

**Figure 5.**
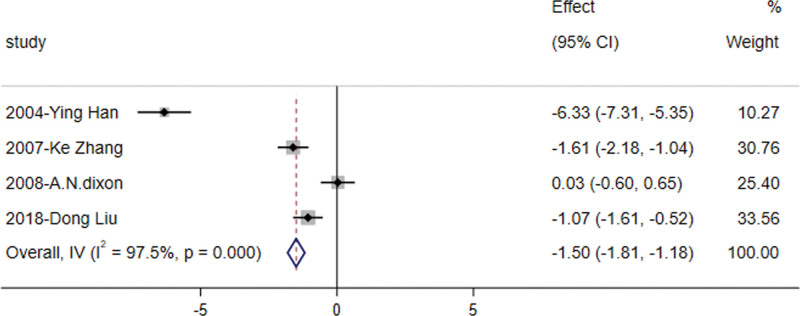
Forest plot of blood glucose levels at 2 hours postprandial in the orlistat intervention versus control group.

The analysis included 4 studies^[10–13]^ involving a total of 265 participants comparing orlistat to control intervention in individuals with IGT at the 2-hour mark after meals. During too high heterogeneity (*I*^2^ > 50%), a random-effects model was used. Orlistat significantly reduced the 2-hourpostprandial glucose level in patients with IGT [RR = −1.497, 95% CI (−1.811, −1.183), z = −9.345, *P* = .000] (Fig. 5), indicating its potential to delay the progression to T2DM and even reverse the normal state of glucose tolerance in patients with impaired glucose tolerance.

#### 3.3.5. Subgroup analysis

**Figure 6.**
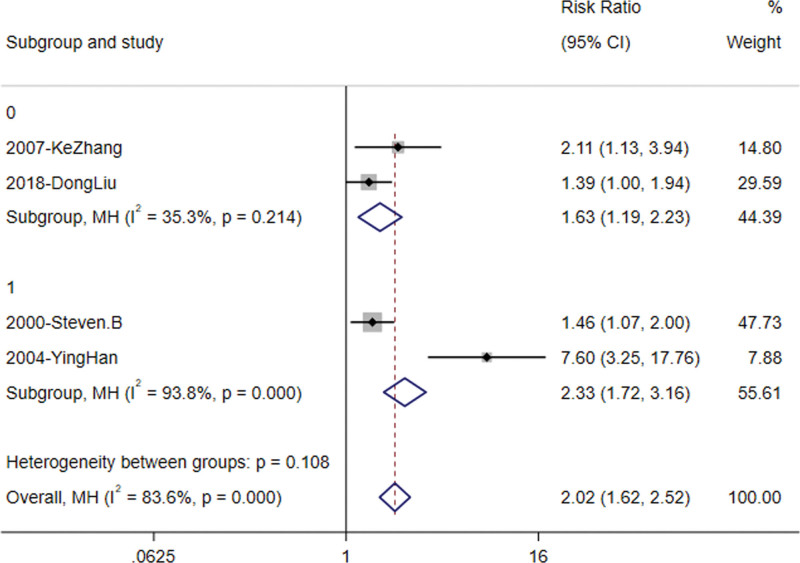
Subgroup analysis of the forest plots.

Using the observation time of 1 year, the study was divided into 2 groups for subgroup analysis. Due to the lack of statistical data on the conversion of prediabetes to diabetic population, it is difficult to calculate *P*-values. This subgroup analysis used inverse analysis to analyze the rate of prediabetic population into normal population. The results of subgroup analysis showed that the intervention time was proportional to the ratio of prediabetes reversal to normal glycemic status: Intervention for <1 year [RR = 1.63, 95 CI% (1.19, 2.23), z = 3.057, *P* = .002], and experiments intervening for 1 year or more [RR = 2.33, 95 CI% (1.72, 3.16), z = 5.453, *P* = .000], and total [RR = 2.02, 95 CI% (1.62, 2.52), z = 6.249, *P* = .000] (Fig. 6). However, at the same time, we can see that the heterogeneity of Ying Han^[10]^ articles is high, so the results of this analysis have certain limitations.

### 3.4. Adverse reactions

Three studies^[9–11]^ have reported adverse effects of orlistat, primarily in the gastrointestinal system. These effects include oily stool and mild to moderate gastrointestinal symptoms that typically resolve after a period of drug use. Additionally, some patients experience a slight decrease in fat-soluble vitamins; however, this has not exceeded the lower limit of normal. It is recommended that oral supplementation during the course of treatment to address this issue. Overall, orlistat has a good long-term safety profile, and there is almost no difference in adverse reaction rates between 2 and 4 years,^[9]^ indicating that orlistat has better long-term safety.

### 3.5. Publication bias

**Figure 7.**
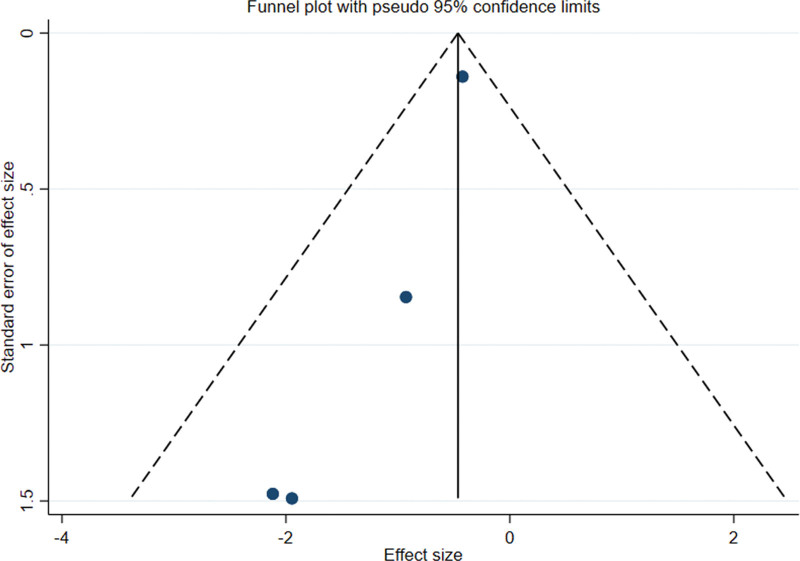
Funnel plot of the orlistat intervention versus the control group.

**Figure 8.**
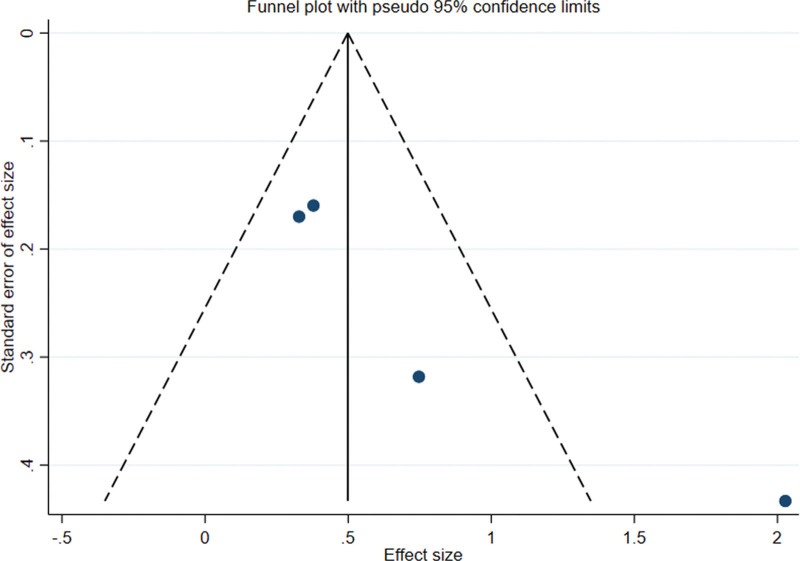
Funnel plot of abnormal glucose tolerance reversal rate of orlistat intervention versus the control group.

Funnel analysis of the included articles using Stata software revealed asymmetry on both sides, indicating publication bias (Figs. 7 and 8). For the following reasons: the small number of references considered for inclusion and the insufficient number of relevant studies may be related to the bias of investigators and journals toward positive results.

## 4. Discussion

The pre-disease stage of T2DM, known as prediabetes, encompassed 3 categories: IGT, impaired fasting glucose or both, among these, IGT constitutes a significant proportion and poses a higher risk for progressing into T2DM.^[14]^ The prevalence of diabetes in China is the highest globally, and projections suggest that by 2045, it will remain so.^[3]^ Diabetes and its complications have emerged as a major public health concern in China, imposing significant financial burdens.^[15]^

One of the primary factors contributing to the significant increase in IGT incidence is the shift in modern dietary patterns,^[16]^ characterized by a preference for high-fat and richly flavored foods with elevated levels of oil and sugar. This has led to disruptions in glucose and lipid metabolism, with up to 37% of untreated prediabetic patients progressing to diabetes^[17]^ within 4 years. Currently, the IGT population skews younger and is popular,^[18]^ yet prediabetes remains insufficiently addressed with low patient awareness rates.^[1]^ The primary intervention for IGT is still centers on lifestyle changes aimed at weight management, controlling dietary energy intake, increasing physical activity levels, etc. However, due to the high workload, irregular diet and lifestyle patterns, as well as low awareness of IGT among individuals, it becomes challenging to modify unhealthy habits, thereby impacting the treatment outcomes for IGT patients.^[19]^ Moreover, achieving weight loss goals through subjective control of diet and exercise proves more difficult; hence there is a particular need to identify suitable medications that can assist in weight management.^[20]^ The compound of orlistat is a potent and specific gastrointestinal lipase inhibitor with long-acting effects. It achieves this by binding to the active serine of gastric lipase and pancreatic lipase in the stomach and small intestine, effectively preventing the hydrolysis of dietary lipids into absorbable free fatty acids and monoacylglycerols. Consequently, it significantly reduces lipid intake.^[21]^ In addition to its lipase inhibitory effects, orlistat can also indirectly modulate appetite^[22]^ by increasing the body’s dopamine levels and inhibiting glutamate neurotransmitters, thereby achieving the objective of weight control. The absorption of orlistat is minimal, with a half-life of 1–2 hours and predominant metabolism occurring in the small intestine wall. Following administration, approximately 97% of orlistat is excreted through feces, while only 2% is eliminated via renal excretion. Due to its extremely low therapeutic dose concentration and negligible pharmacological significance,^[23]^ it exhibits a high level of safety for human use. Additionally, orlistat has also been approved by the US FDA for treating adolescent obesity,^[24]^ further confirming its safety profile.

Orlistat may serve as an efficacious pharmacological intervention for individuals with IGT, particularly in those who are obese, to effectively manage their progression towards diabetes. However, the current lack of relevant evidence necessitated our performance of this meta-analysis to further validate its efficacy.

This article conducted a search for trials of orlistat experiments in individuals with IGT and identified RCT experimental data that met the criteria. A total of 6 RCT articles were included, involving 1076 subjects who all had IGT. This study demonstrated that administering 120 mg of orlistat 2 to 3 times daily can effectively reduce weight in obese patients with impaired glucose tolerance, as well as significantly decrease postprandial and fasting blood glucose levels. Additionally, it regulates their glucose and lipid metabolism to achieve normal levels. Orlistat has demonstrated efficacy in controlling the progression of IGT patients to T2DM, and even facilitating a reversal to normal glucose tolerance. At the same time, one of the experiments^[13]^ confirmed the synergistic effect of orlistat in controlling the progression of prediabetes to diabetes with metformin in controlling its progression. However, due to limited related reports, meta-analysis has not been utilized for safety assessment of orlistat. A comprehensive review of multiple studies indicates that adverse effects primarily manifest as gastrointestinal reactions, which are generally manageable and/or transient with continued use. The long-term safety profile is generally favorable, which aligns Tak YJ conclusions^[25]^; however, there have been reports suggesting a slight potential for hepatotoxicity and nephrotoxicity associated with orlistat usage,^[26,27]^ therefore, necessitating regular monitoring of liver and kidney function during treatment. The inhibitory effect on fat absorption by this inhibitor may also impact the absorption of certain drugs,^[28]^ necessitating consideration of potential drug interactions when co-administered. Adverse effects observed in studies with longer intervention periods are generally consistent with those reported in shorter-term studies, indicating a favorable safety profile for prolonged usage.

Limitations of this study include: ① A small number of RCT experiments were included in the study, and the sample size of subjects was limited, which requires further validation; ② the experimental studies selected in this analysis often had differences in the dose of orlistat used for intervention, but subgroup analysis was difficult due to the small number of included studies; ③ some of the data included in the literature examined in the study exhibit certain limitations, leading to a further reduction in the available dataset. Therefore additional investigation is warranted to explore the efficacy and safety of orlistat in individuals with IGT; ④ the majority of participants mentioned in this article are obese participants, necessitate further explored should be conducted about non-obese people. These aforementioned deficiencies may introduce research bias and thus should be given more attention for subsequent large-sample experiments that are more scientifically rigorous and precise.

## 5. Conclusion

In summary, orlistat effectively reduce the 2-hour postprandial blood glucose and fasting blood glucose levels in patients with IGT, thereby delaying the progression from IGT to T2DM and facilitating the reverse of IGT status to normal glucose tolerance. The majority of adverse events are mild to moderate in severity, most of which resolve spontaneously without treatment. These events primarily affect the gastrointestinal tract, and long-term safety profile is favorable.

## Author contributions

**Data curation:** Zhao Gao, Mengwen Huang.

**Funding acquisition:** Guixiang Ti.

**Investigation:** Jiaxin Wang.

**Methodology:** Pin Lv.

**Resources:** Huihui Jia.

**Software:** Zhao Gao.

**Supervision:** Jing Zeng.

**Writing – original draft:** Zhao Gao.

**Writing – review & editing:** Zhao Gao.
